# Evolutionary dynamics of *Enterococcus faecium* reveals complex genomic relationships between isolates with independent emergence of vancomycin resistance

**DOI:** 10.1099/mgen.0.000048

**Published:** 2016-01-19

**Authors:** Sebastiaan J. van Hal, Camilla L. C. Ip, M. Azim Ansari, Daniel J. Wilson, Bjorn A. Espedido, Slade O. Jensen, Rory Bowden

**Affiliations:** ^1^​ Department of Microbiology and Infectious Diseases, Royal Prince Alfred Hospital, Sydney, NSW, Australia; ^2^​ Antibiotic Resistance & Mobile Elements Group, Ingham Institute for Applied Medical Research, Sydney, NSW, Australia; ^3^​ Oxford Genomics Centre, Wellcome Trust Centre for Human Genetics, University of Oxford, Oxford, UK; ^4^​ Oxford Martin School, University of Oxford, 34 Broad Street, Oxford, UK; ^5^​ Nuffield Department of Medicine, University of Oxford, Oxford, UK; ^6^​ Molecular Medicine Research Group, School of Medicine, University of Western Sydney, Sydney, NSW, Australia

**Keywords:** *Enterococcus faecium*, infection control, multi-locus sequence typing, recombination, transposon, vancomycin resistance

## Abstract

*Enterococcus faecium*, a major cause of hospital-acquired infections, remains problematic because of its propensity to acquire resistance to vancomycin, which currently is considered first-line therapy. Here, we assess the evolution and resistance acquisition dynamics of *E. faecium* in a clinical context using a series of 132 bloodstream infection isolates from a single hospital. All isolates, of which 49 (37 %) were vancomycin-resistant, underwent whole-genome sequencing. *E. faecium* was found to be subject to high rates of recombination with little evidence of sequence importation from outside the local *E. faecium* population. Apart from disrupting phylogenetic reconstruction, recombination was frequent enough to invalidate MLST typing in the identification of clonal expansion and transmission events, suggesting that, where available, whole-genome sequencing should be used in tracing the epidemiology of *E. faecium* nosocomial infections and establishing routes of transmission. Several forms of the Tn*1549*-like element–*vanB* gene cluster, which was exclusively responsible for vancomycin resistance, appeared and spread within the hospital during the study period. Several transposon gains and losses and instances of *in situ* evolution were inferred and, although usually chromosomal, the resistance element was also observed on a plasmid background. There was qualitative evidence for clonal expansions of both vancomycin-resistant and vancomycin-susceptible *E. faecium* with evidence of hospital-specific subclonal expansion. Our data are consistent with continuing evolution of this established hospital pathogen and confirm hospital vancomycin-susceptible and vancomycin-resistant *E. faecium* patient transmission events, underlining the need for careful consideration before modifying current *E. faecium* infection control strategies.

## Data Summary

1. Genome read data for all samples has been deposited into European Nucleotide Archive NA under accession number PRJEB8624 (http://www.ebi.ac.uk/ena/data/view/PRJEB8624)
2. Annotated Plasmid pJEG050 has been deposited into GenBank under accession number KR066794 (http://www.ncbi.nlm.nih.gov/nuccore/827342679/)

## Impact Statement

*Enterococcus faecium* has been identified as one of six key pathogens that are a threat to future healthcare provision. This is due to its ability to cause devastating infections in susceptible patients, which are difficult to treat as a result of *E. faecium* acquiring antimicrobial resistance, especially to first-line therapy such as vancomycin. Not surprisingly, hospitals implement several interventions to prevent spread of this organism especially if it is vancomycin-resistant. However, the utility of this strategy is unknown because of suboptimal understanding about the evolutionary dynamics of this pathogen. We present a comprehensive analysis of the largest dataset to date of *E. faecium* from a single institution and also examine the context of our isolates in relation to other Australian isolates. Not only do we document the genomic plasticity we are able to show examples of recombination leading to novel MLST emergence and recreation of old MLST types. Sources of recombination are identified with further hospital adaptation documented. Antimicrobial resistance dynamics are explored with patient transmission events observed. Overall our results substantially add to the current debates around *E. faecium* infection control and will assist in forming policies of how to curb the ongoing spread of *E. faecium.*

## Introduction

Enterococci form part of the normal gastrointestinal flora of healthy humans. Although two species, *Enterococcus faecalis* and *Enterococcus faecium*, predominate, the clinical impact of *E. faecium* as a nosocomial pathogen has increased substantially and risen to prominence over the last two decades (Arias & Murray, 2012; Hidron *et al.*, 2008). Infections caused by *E. faecium* are difficult to treat, because of both intrinsic and acquired resistance to a variety of antibiotics including vancomycin, regarded currently as first-line therapy (McGowan & Tenover, 2004). Consequently, vancomycin-resistant *E. faecium* (VRE) infections are associated with substantially higher patient morbidity and mortality compared with vancomycin-susceptible (VSE) infections (DiazGranados *et al.*, 2005).

Resistance to vancomycin in *E. faecium* is generally conferred by either the *vanA* operon, predominant in the United States (Arias & Murray, 2012; Courvalin, 2006), or the *vanB* operon common in Australia (Coombs *et al.*, 2014), almost always carried on the transposable element Tn*1549* (or a closely related derivative) (Garnier *et al.*, 2000). VRE first emerged in Europe in the 1980s (Werner *et al.*, 2008), and since then has been detected worldwide (Ramsey & Zilberberg, 2009; Willems *et al.*, 2005) with steadily increasing incidence in numerous countries including Australia. In recognition of the continuing threat it poses to healthcare delivery, *E. faecium* was named as one of six key problem or ESKAPE bacteria by the Infectious Diseases Society of America (Boucher *et al.*, 2009).

The ability to easily acquire additional genetic elements including antimicrobial resistance determinants may in part explain the rise of *E. faecium* as a nosocomial pathogen (Arias & Murray, 2012). Moreover, high levels of homologous recombination have been linked to the emergence of specific hospital-adapted clones (e.g. the group designated MLST clonal complex 17) with the vast majority of infection isolates clustering within clade A1 (Lebreton *et al.*, 2013). Consequently, genome plasticity has been cited as evidence for the recent and ongoing evolution of *E. faecium* (de Been *et al.*, 2013; Howden *et al.*, 2013; Willems *et al.*, 2012) with clones thought to have received at least some genetic material from (colonizing) *E. faecium* isolates within the patient or the environment through cross-transmission (de Been *et al.*, 2013). However, apart from the *vanB* operon, the identities, specific functions and overall contribution of imported sequences to a healthcare-associated phenotype remain poorly understood, reflecting perhaps the difficulty of assembling informative isolate collections and difficulties in determining specific recombination events. Although previous studies have reported signals for ongoing evolution, especially in favour of disease-causing clones (de Been *et al.*, 2013; Willems *et al.*, 2011, 2005), none has examined the alternative hypothesis of new clone emergence as a result of continuous recombination.

In locations where VRE has become endemic much effort is put into preventing cross-transmission by screening at-risk patients and isolating VRE-colonized patients. Although these are logical responses to a serious problem, the effectiveness of these costly strategies is difficult to test objectively for several reasons: they are applied only where VRE infection is already a problem, they may be difficult to disentangle from changes in antimicrobial usage, and absolute infection rates may not be informative about the sources and transmission patterns of VRE in hospitals. One remedy for the lack of resolution provided by headline rates of infection has been the use of genetic information in the form of MLST to assess relationships between isolates and infer outbreaks. MLST remains the *de facto* standard for identifying outbreaks despite the potential availability of whole-genome sequencing (WGS).

To better understand the dynamics of vancomycin resistance and its relationship to genome micro-evolution in a well-delineated population, we assembled and analysed whole-genome sequences of a substantial and comprehensive single-hospital collection of bloodstream *E. faecium* isolates with a high proportion of VRE. We used this information to investigate the role of recombination in the within-hospital evolution of the organism, including the emergence of vancomycin resistance, and to assess the overall relationships to external (other Australian) *E. faecium* isolates, which will further inform the current debates about the likely effectiveness of standard approaches for VRE infection control.

## Methods

### Isolate collection

In total, 146 consecutive non-duplicate blood culture isolates from patients with confirmed *E. faecium* bacteraemic infection at a single institution, Royal Prince Alfred Hospital (RPA) in Sydney, Australia, from two distinct time intervals (2004–2007 and 2011–2013), were identified for study (Table S1, available in the online Supplementary Material). Of these, seven isolates had not been stored and seven were later excluded because of poor sequence coverage linked to suboptimal DNA extraction, leaving 132 isolates representing 90 % of identified *E. faecium* bloodstream infections at RPA over the two time periods. Initial *E. faecium* isolation from flagged positive blood-culture bottles included subculturing onto horse-blood agar (HBA) followed by species confirmation by routine methods, susceptibility testing using Vitek2 (bioMérieux) and *vanB* PCR to confirm phenotypic vancomycin resistance (Adams, 2006).

### Sequencing and assembly

Genomic DNA samples were sequenced on the Illumina HiSeq 2500 platform using 151b paired-end TruSeq chemistry. Reads were mapped to the Aus0085 genome (2994661 bp, GenBank: CP006620, an ST203 *vanB* VRE isolated from a patient in Melbourne, Australia, in 2009) (Lam *et al.*, 2013), using Stampy v1.0.23 (Lunter & Goodson, 2011) with an expected substitution rate of 0.01. Variants were called using SAMtools v0.1.19 (Li *et al.*, 2009) and filtered for read depth (minimum 20), read base quality (minimum Phred score 20) and mapping quality (minimum 30). Variation at indels and in the presence of mobile elements was excluded from the mapping-based analysis.

### MLST, resistome analysis and transposon assembly

*De novo* assemblies constructed using Velvet v1.2.10 (Zerbino, 2010) with VelvetOptimizer v2.2.5 (http://bioinformatics.net.au/software.velvetoptimiser.shtml) were used for *in-silico* MLST (http://pubmlst.org/) and to detect *vanB* sequences using blastn v2.2.28 against the Aus0085 genome. Susceptibility and *vanB* PCR retesting in two isolates whose recorded phenotype and inferred genotype were discordant confirmed WGS accuracy. Tn*1549*-like sequences carrying the *vanB* locus were identified by comparing *de novo* contigs against complete Tn*1549* sequences from Aus0085 (GenBank: CP006620 nucleotide positions 723825–781312) and Aus0004 (GenBank: CP003351 nucleotide positions 2835430–2869240) *E. faecium* genomes. Obtained sequences were aligned using progressiveMauve (Darling *et al.*, 2010) and annotated using CLC Genomics Workbench v7.5.1 (CLC bio, Qiagen) with isolate-specific insertion sites identified relative to Aus004.

### Recombination

Homologous recombination was assessed using an iterative application of ClonalFrameML (Didelot & Wilson, 2015) as follows. (1) A maximum-likelihood input phylogeny, rooted at the midpoint, was inferred using RaxML v7.0.43 (Stamatakis, 2006) under the General Time-Reversible model, and analysed using ClonalFrameML. (2) Identified recombination intervals were masked in isolates containing the recombination event or descending from the branch containing the event. The modified dataset was then used to generate a new RaxML input phylogeny. (3) ClonalFrameML was rerun with the new phylogeny and the original (unmasked) genome alignment. Steps 2 and 3 were repeated until the number of recombination events and phylogeny remained stable. Estimates of recombination parameters and the set of recombination events were recorded from the final run.

### Genomic context

All Illumina reads from another Australian study (GenBank: PRJNA205886) (Howden *et al.*, 2013), strain DO (GenBank: NC_017960) and Aus0004 (GenBank: CP003351) were analysed using the above pipeline to reconstruct a phylogeny of available Australian isolates.

### Visualization of phylogenetic trees and recombination events

Trees were visualized and manipulated with Figtree v1.4.2 (http://tree.bio.ed.ac.uk/software/figtree).

### Nucleotide sequence accession number

The sequence reads, mapping-based variant calls and *de novo* assemblies for all isolates are available from ENA under accession number PRJEB8624.

## Results

### Sequencing and the impact of recombination

A total of 2.4 Mbp of aligned sequence, comprising 81 % of the Aus0085 reference, 2703 genes and 21288 single nucleotide variants (SNVs) (reflecting the large species-wide diversity captured), was called unambiguously in all 132 successfully sequenced isolates (Table S1) of which 49 (37 %) carried the *vanB* operon and were vancomycin-resistant.

**Fig. 1.**
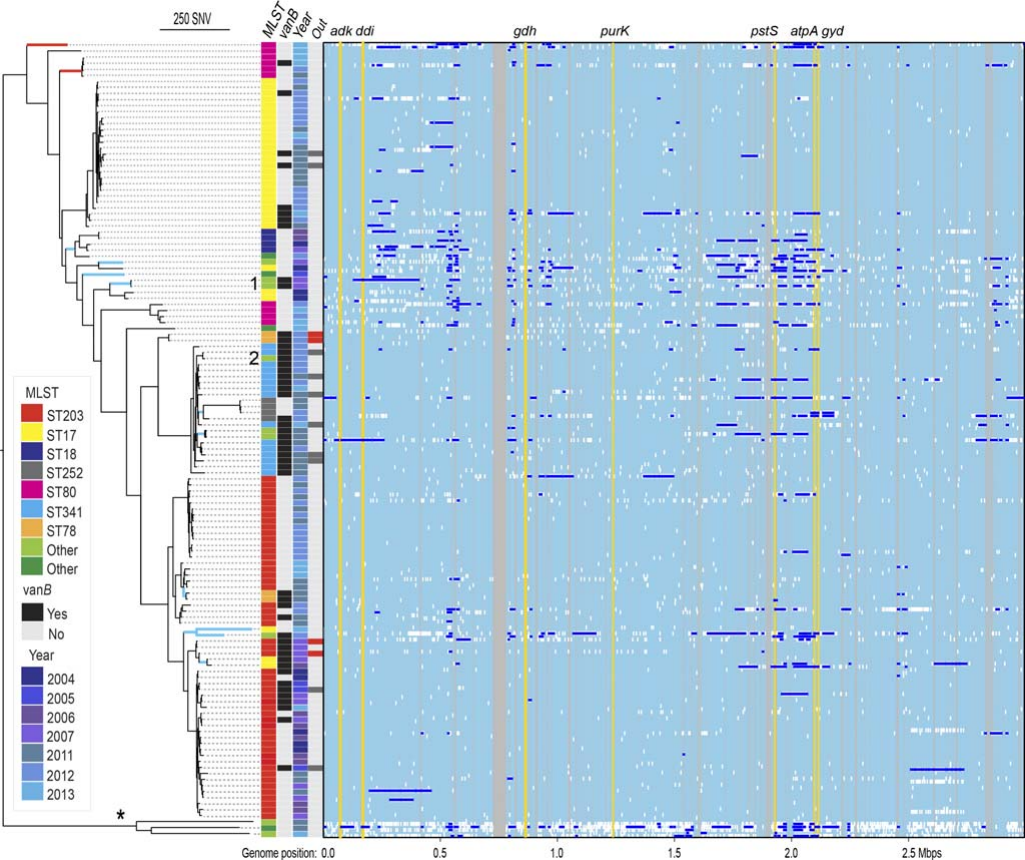
Clonal phylogeny and inferred recombination events. Midpoint-rooted clonal phylogeny of 132 *E. faecium* bloodstream isolates; metadata (centre) and recombination events (right), estimated jointly as described in Methods. The sizes and genomic locations of recombination fragments (dark blue line segments) and the positions of SNVs (white ticks) occurring along branches in the phylogeny are aligned with branches in the phylogeny. Vertical grey lines mask genomic fragments with incomplete data and vertical yellow lines show the positions of seven MLST loci. Despite the use of a plausible statistical model for recombination, marked clusters of substitutions remain in the estimated clonal phylogeny, suggesting additional undetected imports and emphasizing the analytical difficulty in distinguishing real divergence (long branches) from imports. Branch with asterisk indicates three divergent, non-CC17 isolates. Turquoise and red branches carry recombination events directly affecting ST, i.e. MLST loci leading to false negative and false positive MLST clustering, respectively. Isolate meta-data are depicted from left to right: sequence type, *vanB* vancomycin resistance status, year of isolation and epidemiological assignment to a putative outbreak (Out) by MLST. ‘Other’ STs use alternating green shades for convenience for successive distinct STs. Novel ST types ST990 (Number 1) and ST991 (Number 2) are indicated. Isolates assigned to putative MLST defined outbreaks are depicted by the grey bars with evidence of two definitive transmission events (red bars) when using WGS (see text and Table S3 for further details).

Using ClonalFrameML (Didelot & Wilson, 2015) as described in Methods, we detected 686 imports of homologous sequences among the 132 isolates. Recombination events were scattered across the clonal phylogeny, clustered on the genome (Fig. 1) and varied widely in size, from near the practical lower limit of detection (39 bp) to >100 kbp (six events: largest 258 kbp; 10.7 % of shared genome) (Fig. S1). The ratio of the estimated contributions of homologous recombination and mutation to observed substitutions (i.e. r/m) across the overall phylogeny was 6.1 (the ratio of estimates for R and θ – recombination and mutation events – was 0.22), moderately high among estimates for collections of bacterial samples representing successive cases in a host population. This number represents a credible increase compared with previous estimates (Vos & Didelot, 2009) (due to expanded and denser population sampling and updated methodology), but is likely to be an underestimate as some recombination events remain difficult to detect (Fig. 1).

### Epidemiology, MLST and outbreak investigations

The reconstruction of clonal relationships allows us to assess the epidemiology of *E. faecium* in a single hospital, and the effectiveness of MLST (which compares the sequences of seven gene fragments of ∼500 bp between isolates), in capturing those patterns.

*In silico* MLST places 126 (95 %) of the 132 isolates, including 51 sequence type 203 (ST203) isolates, in the well-established clonal complex 17 (CC17). Two new STs (two ST990 and one ST991) were determined (Fig. 1; MLST locus details in Table S2) which could also be added to CC17. Quite apart from its ∼1000-fold lower resolution of micro-evolutionary variation compared with WGS, the integrity of MLST-based classification compared with reconstructed clonal relationships was seriously affected by recombination. Some 28 inferred recombination events overlapped at least one (*ddl*, *atpA*, *gyd*, *pstS*) of the seven MLST loci (Fig. 1). Sequence types were not monophyletic in either the naı¨ve or the corrected clonal phylogeny, with several examples of distinct groupings by time and MLST type (e.g. ST203 in 2011 and ST17 in 2013; Fig. 1). In fact, an inferred recombination changed the ST within a clonal cluster at least ten times (e.g. ancestral ST203 and ST341, to ST17 and ST252, respectively) leading to potential false negative clusterings (Fig. 1; turquoise branches). In one of these events (Fig. 1; indicated by the number 1), a recombination event across a single locus (*atpA)* resulted in the emergence of a new ST, 990, in two isolates and confirms the alternative hypothesis of new clone emergence as a result of continuous recombination. Conversely, one possible false-positive clustering resulted from the recreation of an already present ST (Fig. 1; red branches).

These observations when combined with clinical–epidemiological data would have resulted in incorrectly missing or assigning *E. faecium* outbreaks. Similarly, when examining the seven potential VRE outbreaks (defined as >1 VRE bacteraemia of the same MLST type within the same calendar month) (Fig. 1 and Table S3), two events (outbreaks coloured red in Fig. 1) would be confirmed by WGS when using a cut-off of 10 SNV differences (nominally 1 year's divergence at a molecular clock rate of ∼10 SNVs per genome per year) (Howden *et al.*, 2013; Lebreton *et al.*, 2013).

These observations and robust evidence for recombination across housekeeping genes lead us to conclude that MLST is neither sensitive nor accurate enough for *E. faecium* molecular epidemiology. By comparison, the enhanced resolution of WGS would probably also enhance the early detection of new clonal expansion events (Miller *et al.*, 2014).

### Donors and recipients of recombined sequences

Our dataset of 132 geographically and temporally related *E. faecium* genomes was investigated as the primary plausible source(s) of sequence imports for other genomes in the dataset. A non-descendant genome was considered a plausible within-species source of an imported fragment of ≥ 200 nt if it contained the most similar sequence >99.9 % identical to and overlapping at least 90 % of the imported fragment (identically matching sequences were considered as equally likely sources). Where there was no match within the dataset, plausible sources were identified in the GenBank nucleotide database.

The potential donor of inferred imports could be ascribed to isolates sequenced within the dataset for 80.5 % (552 of 686) of all events, where at least one exact match or a plausible match was inferred for 481 and 71 events, respectively. Searches of the remaining 108 imported fragments ≥ 200 nt using blastn identified *E. faecium* (but not an isolate from the current study) as the likely source for all remaining events and placed the lower bound for recent extra-species imports at zero (provided either sequence imports into our dataset have not occurred more than once or closely related lineages have not co-inherited the same recombination sequence). The lack of direct evidence favouring a role for closely related non-*E. faecium* enterococcal species in acquisition of genetic material by *E. faecium*, however, does not exclude a more distant origin for some of these identified recombinant sequences and also does not exclude a shuttle role for other commensal species in the supply of new material. Nevertheless, the numerous within-species recombination events suggest the importance of other *E. faecium* lineages in the overall adaptation of hospital-adapted CC17 isolates.

### Distribution of recombination across the genome

Of the 2703 annotated genes in the shared genome, 1880 (70 %) underwent at least one recombination event, with several regions of the shared genome seemingly overrepresented (Fig. S2). No particular Clusters of Orthologous Group categories (including antimicrobial resistance) were enriched among these highly replaced loci, failing to support one possible simple explanation for recombination heterogeneity along the genome (Croucher *et al.*, 2015). Nor were there obvious patterns of replacement in the *esp* (enterococcal surface protein) gene which encodes a virulence factor associated with hospital adaptation (Coque *et al.*, 2002). All CC17 and none of the non-CC17 isolates (branch denoted by the asterisk in Fig. 1) in our dataset carried *esp*, consistent with its acquisition by an ancestor of present-day CC17 and lending support for the importance of this gene in hospital adaptation.

### Vancomycin resistance and Tn*1549*

**Fig. 2.**
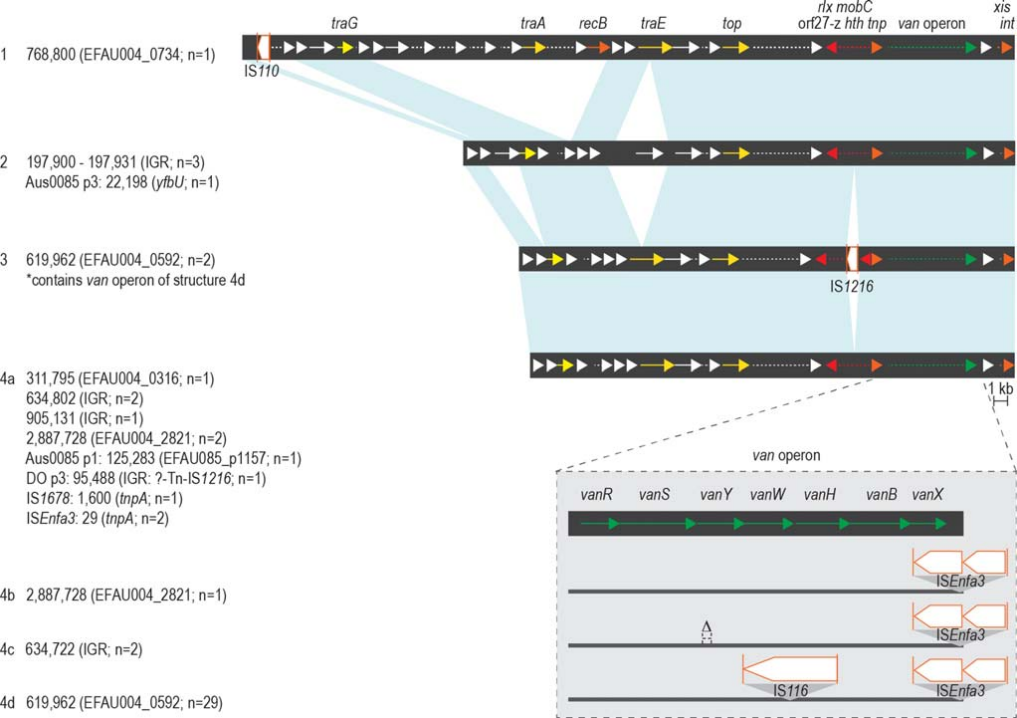
Tn*1549-*like *van*-carrying transposons. Gene arrangements of representative Tn*1549-*like transposons and their included *vanB* operons (inset). Labels show the numbers of isolates sharing insertion positions [with respect to the Aus0004 genome for comparison with Howden *et al.* (2013) – sharing is consistent with a single ancestral insertion at each position] and the insertion locus [gene, hypothetical ORF, or intergenic region (IGR)]. Transposon structure 2 was found in two different sites within a 32 bp region of an IGR. Where the transposon inserted into a mobile element, the name of the plasmid or IS is given; the nucleotide position of the insertion is given with respect to the reference plasmid sequence or the transposase gene of the IS. Coloured arrows indicate: red, mobilization genes; orange, transposon-related genes; green, vancomycin resistance genes (see inset); white, hypothetical ORFs; yellow, annotated genes not in one of these categories. IS elements (white pentagons) are shown with their insertion positions.

Four main *Tn1549*-like transposon structures carrying *vanB* were distinguishable (Fig. 2) among the 49 vancomycin-resistant isolates, including one with alternative secondary variants involving insertions of extra sequences into the *vanB* operon. A temporal relationship among circulating transposon structures was observed with Tn*1549* structure 4d largely replacing structure 4a as the dominant form from 2011 onwards.

**Fig. 3.**
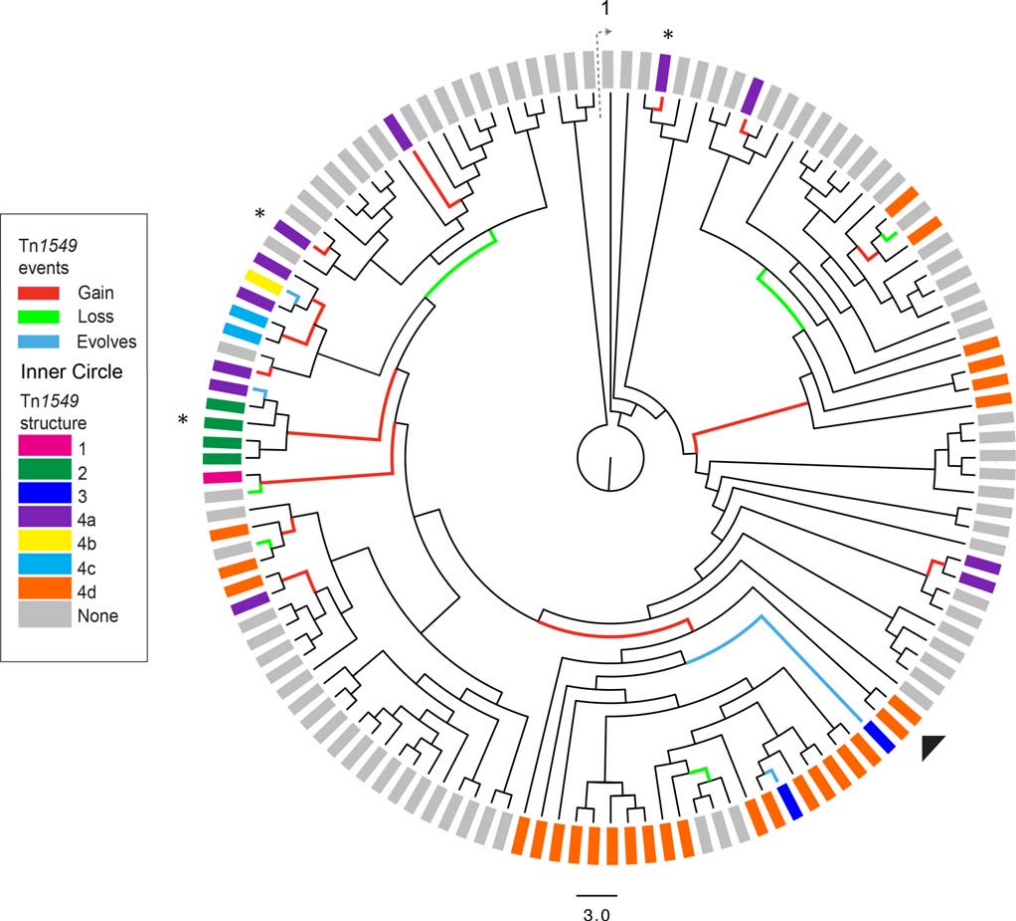
*vanB* Tn*1549*-acquisition/loss and transposon evolution events. Annotated circular phylogeny of 132 *E. faecium* isolates. Branch colours represent a parsimonious reconstruction of *vanB* transposon acquisition and loss events based on transposon structure and insertion site (green, gain; red, loss; blue, evolution of transposon *in situ*; from previous acquisition). Inner circle colours reflect Tn*1549* structure (see Fig. 2 for more details). The number depicts the starting point of the tree relative to Fig. 1. Asterisks indicate plasmid-borne transposon. The triangle indicates two isolates that on the core genome level are identical and carry the same Tn*1549* consistent cross transmission.

In three separate isolates, Tn*1549* was detected on a distinct plasmid (Fig. 3, denoted by the asterisks): in one isolate, Tn*1549* was inserted in the *yfbU* gene of an Aus0085 p3-like plasmid designated pJEG050 (GenBank accession no. KR066794; Fig. S3). In the other two isolates, transposons were found inserted in previously described enterococcal plasmids (Aus0085 p1, GenBank accession no. CP006621; DO p3, GenBank accession no. CP003586). The detection of several Tn*1549*-carrying plasmids indicates the importance of extra-chromosomal elements in the transfer of resistance (Woodford *et al.*, 1995), although the relative impact of this and other mechanisms of transmission remains uncertain.

For the remaining 46 isolates, Tn*1549* was chromosomally integrated. Insertion sites occurred at least 41 kb from areas of extensive recombination within the shared genome, excluding a mechanistic link between transposition and recombination. Transposons of the same structure tended to insert at the same chromosomal location, but this was not always the case (structure 4a, Fig. 2). Once the clonal phylogeny was accounted for, rather than a literal interpretation of insertion sites, there was no evidence of repeated insertion into any ‘hotspot’ position.

Vancomycin-resistant isolates were scattered across and in distinct branches of the clonal phylogeny, implying at least 15 distinct *vanB*-carrying transposon acquisition and six loss events in the history of the samples (Fig. 3). In addition, taking into account the phylogeny unmasked four *E. faecium in situ* transposon adaptation events. This pattern of gains and losses was confirmed when temporal isolate data were included and is best illustrated by isolates clustering within MLST ST341, which were collected in 2011 and 2012, all harboured the same Tn*1549* substructures (4d; Fig. 3).

Notably, all detected transposon gain events occurred in isolates closely related or identical to sampled VSE isolates, consistent with the suggestion that VRE emerges from the circulating enterococcal population followed by VRE transmission with definitive evidence for one such transmission with two temporally linked isolates, identically matched at the core genome level and Tn*1549* (triangle in Fig. 3).

### Genomic context

**Fig. 4.**
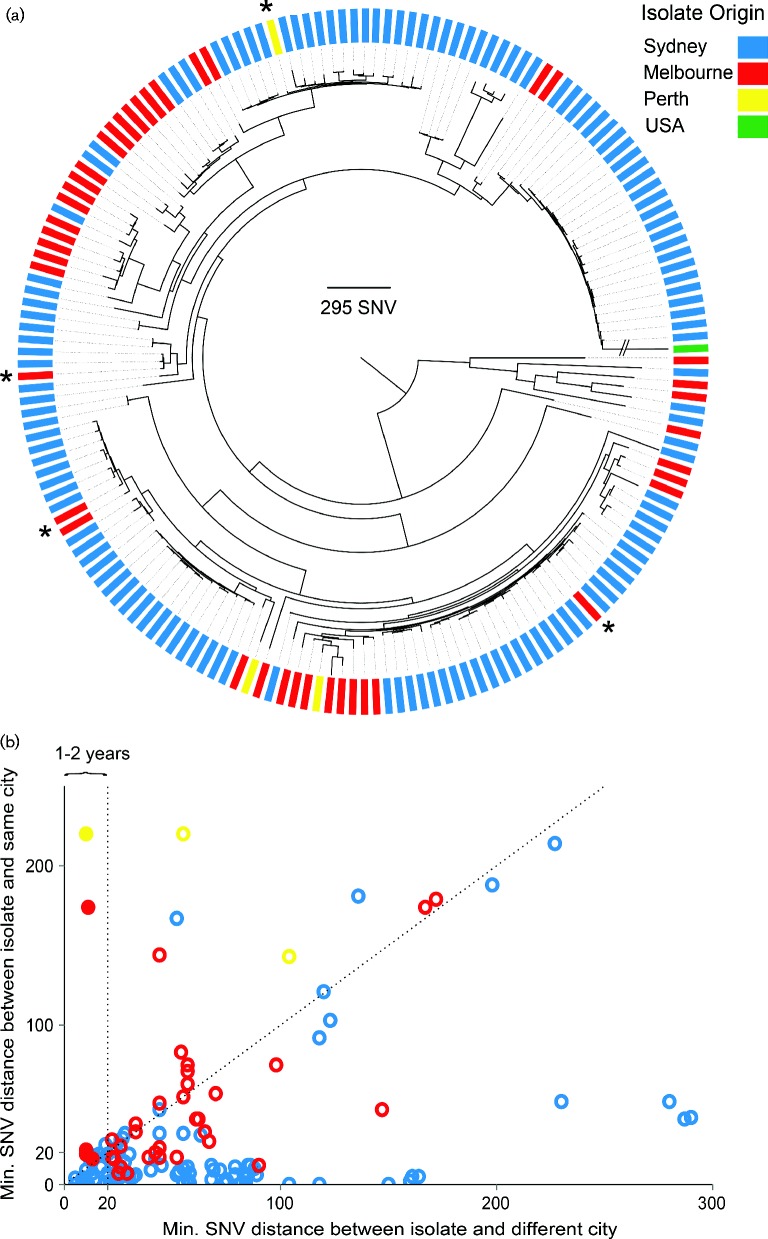
Australian phylogeny. (a) Circular clonal phylogeny of 177 *E. faecium* isolates comprising 132 bloodstream isolates from a single institution in Sydney (current study) and 45 isolates (42 from Melbourne, coloured red, and three VRE isolates from Perth, coloured yellow) from Howden *et al.* (2013). The same *E. faecium* reference strain DO (a VSE ST18 from the USA, 1998) was included. Note that the branch for strain DO has been shortened more than fourfold, as indicated by //. (b) Each isolate [coloured by city as in (a)] is plotted as the mutational distance in SNV to the most similar isolate from the same city (*y*-axis) or from a different city (*x*-axis). Points near the *x*-axis (below the diagonal) represent the majority of isolates, whose nearest neighbour is in the same population; points above the diagonal represent isolates with a nearest neighbour in another population; and points near to the diagonal (*x* = *y*) may be interpreted as isolates in lineages (at different scales of divergence) that are shared between cities. Points plotted as filled circles correspond to the same isolates marked with asterisks as in (a), and provide some evidence for short-term inter-city spread. Seven isolates that were only distantly related to other isolates are not plotted. The vertical dotted line represents approximately 1–2 years between isolates.

To place our results in context, we examined the genomic relationships between isolates from single hospitals in Australia's two largest cities, Sydney (this study) and Melbourne, 700 km apart, plus three isolates from Perth, a relatively distant state capital (Howden *et al.*, 2013). We obtained read data for all isolates sequenced on the Illumina platform, and analysed them using our framework to generate an enlarged phylogeny (Fig. 4a). We verified the distant relationship between Australian isolates and the reference strain DO (an ST18 isolate from the United States) and revealed a complex relationship between Australian isolates, with interspersed study-specific clustering of samples. Inspection of the clonal phylogeny in conjunction with temporal signals of genetic clustering within our hospital (Fig. 1) suggests a distinct common source or a history of progressively spreading institution-specific sublineages. We defined an arbitrary criterion for very close genomic and epidemiological relationships of ≤ 20 mutational substitutions to identify five highly probable inter-city transfer events (Fig. 4). Of these, four were suggestive of Melbourne–Sydney transfer events with the Melbourne isolates differing from Sydney isolate(s) by fewer than 20 SNVs. The remaining event represented a possible Perth–Sydney transfer event. More generally, we noted that patterns of genomic diversity among *E. faecium* isolates in different Australian centres were not noticeably different but seem to have arisen from a common ancestor. Although this observation does not necessarily imply that the epidemiology (and role of infection control) of *E. faecium* disease in each centre is the same, it seems likely that apparent qualitative differences between centres could result from differences in sample size and sampling patterns.

## Discussion

Our appraisal of the extent of homologous recombination and dissection of the rapid structural evolution of Tn*1549*, carrier of the vancomycin resistance-conferring *vanB* operon, underlines the organism's well-known genomic plasticity (de Been *et al.*, 2013; Lebreton *et al.*, 2013; Willems *et al.*, 2005) placing it towards the high end of the spectrum of the relative effects on molecular variation of homologous recombination and mutational substitution (Chewapreecha *et al.*, 2014). By including a relatively large number of isolates, our study has clearly identified the effects of recombination at the whole-organism level, from an explicit analysis of recombination to an analysis of the recombination gene pool, with implications for our understanding of *E. faecium* evolution.

Dealing in a principled way with recombination via reconstruction of recombination events and revision of the clonal phylogeny has allowed a critical reassessment of the utility of MLST in identifying outbreaks. We conclude that although sequence types previously may have been a useful descriptor, for newly emerging clones and local populations, the ongoing and rapid nature of homologous recombination in *E. faecium* means that MLST is suboptimal in settings where WGS is available (Howden *et al.*, 2013; Willems *et al.*, 2012).

In a widely accepted model, several factors contribute to the success of *E. faecium* and VRE in the hospital environment. Empiric broad-spectrum antibiotic therapy selects for enteric carriage of *E. faecium* due to the organism's intrinsic resistance to several antibiotics. The increased dominance of *E. faecium* (rather than *E. faecalis*) within the gastrointestinal flora subsequently results in enhanced cross-transmission via surfaces and inter-personal contacts (Arias & Murray, 2012) and invasive disease in a subset of colonized patients. This process is augmented by the hospital environment, which in turn has selected for specific *E. faecium* lineages such as CC17 that now predominate among hospital-acquired disease isolates but not among community isolates. Within this model, several questions have been considered, such as: what genetic alterations may have led to the emergence of dominant lineages, and what is the role of *de-novo* acquisition rather than transmission of vancomycin resistance in VRE epidemiology?

More recently, awareness has grown that VRE epidemiology is not as simple as the formation of VRE clones by *vanB* acquisition followed by their transmission (Howden *et al.*, 2013). This has given rise to debates about the utility of current infection control strategies in controlling VRE, with some experts suggesting discontinuing infection control measures all together (Karki *et al.*, 2015). It is important, however, not to consider the observed repeated *de novo* acquisition of vancomycin resistance as failure but rather the consequence of effective classical infection control measures, especially given the observation of a direct case-to-case transmission and genetically clustered VRE (consistent with chains of successive asymptomatic colonization events linking several patients).

Which strategies are most effective and should be employed to control VRE remains uncertain; for instance our data, with its evidence for multiple gains and losses of resistance, suggest that for all its importance in mortality, vancomycin resistance is less crucial to the biology of *E. faecium* at the population level and is independent of core genome adaptation to the hospital environment. In contrast, the dynamics of *E. faecium* seem well established, with selection of specific adaptive traits through genome plasticity leading to the formation and expansion of successful clones within the hospital. Understanding these dynamics in conjunction with those factors, which enable *E. faecium* to gain mobile genetic elements including vancomycin resistance, may unlock the secrets that allow successful containment of VRE. To achieve this, in our view, we will need to better characterize connections between *E. faecium* colonization and infection and substantially improve our understanding of the biology of Tn*1549*–*vanB* within commensals and the greater *E. faecium* population.

To address these important questions, further studies are required that include multiple different sources of isolates allowing for better characterization of the overall hospital VRE burden and the possible extent that transmission adds to this burden. As multiple rounds of gain and loss in non-selective circumstances (e.g. carriage within the hospital) must separate many of the infections, it is clear that future studies should include asymptomatic carriage and mild disease isolates in order to ‘join the dots’, both epidemiologically and in establishing how resistance and resistance-carrying organisms move around the hospital.

*E. faecium* is a highly recombining, multi-resistant organism, which appears both to have initially adapted to the hospital environment and to have repeatedly evolved new clones that have competed with each other, while frequently gaining and losing vancomycin resistance. WGS in an institutional population study of disease cases has allowed us to demonstrate that classical genetic typing is unreliable and unsuitable for routine outbreak tracing where WGS is available, especially given the highly complex genomic relationships observed among isolates within and between institutions. For example, we emphasize the clear genomic clusters into which many isolates fall, finding evidence for cross transmission. In addition, VRE epidemiology may be institution-specific and is likely to be highly dynamic given the genome plasticity observed, suggesting an ongoing need for larger scale surveillance and carefully crafted genomic studies. Such studies will need to include carriage and other non-disease isolates to better determine the epidemiology of within-hospital *E. faecium* and VRE infections and the dynamics of vancomycin resistance acquisition. Together, these insights would support the development of optimized infection control strategies and interruption of transmission limiting the impact of these infections.

## Supplementary Data

1048 Supplementary Data
