# Supplementary material for: Evolutionary dynamics of Enterococcus faecium reveals complex genomic relationships between isolates with independent emergence of vancomycin resistance
Source: Microb Genom. 2016 Jan 19;2(1):e000048. doi: 10.1099/mgen.0.000048 (PMC5049587; doi:10.1099/mgen.0.000048)
Supplement: Supplementary file 1 — Supplementary Data [file mgen-02-48-s001.pdf]

Data ID	Sample Name	Isolate Source	Year of Isolation	Phenotype vancomycin resistant	vanB PCR	Read count	Reference positions recovered	Reference % recovered	In silico MLST	Tree position
wtchgD00002068	Efm0222	Blood culture	2013	No	Negative	4161172	2339764	78.13	ST080	1
wtchgD00002084	Efm0233	Blood culture	2013	No	Negative	3415324	2254405	75.28	ST018	2
wtchgD00002114	Efm0230	Blood culture	2013	No	Negative	4232738	2309789	77.13	ST017	3
wtchgD00002100	Efm0232	Blood culture	2013	Yes	Positive	5456810	2354970	78.64	ST080	4
wtchgD00002064	Efm0215	Blood culture	2012	No	Negative	5564376	2322215	77.55	ST080	5
wtchgD00002101	Efm0157	Blood culture	2011	No	Negative	4191410	2312592	77.22	ST080	6
wtchgD00002070	Efm0197	Blood culture	2012	No	Negative	5321582	2281931	76.20	ST017	7
wtchgD00002134	Efm0172	Blood culture	2011	No	Negative	4276814	2280932	76.17	ST017	8
wtchgD00002072	Efm0209	Blood culture	2012	Yes	Positive	5150118	2311596	77.19	ST017	9
wtchgD00002033	Efm0213	Blood culture	2012	No	Negative	3951560	2240736	74.82	ST017	10
wtchgD00002108	Efm0194	Blood culture	2013	No	Negative	4369090	2280619	76.16	ST017	11
wtchgD00002119	Efm0182	Blood culture	2012	No	Negative	3752030	2278879	76.10	ST017	12
wtchgD00002092	Efm0193	Blood culture	2011	No	Negative	3748684	2270341	75.81	ST017	13
wtchgD00002159	Efm0187	Blood culture	2012	No	Negative	3719582	2271898	75.86	ST017	14
wtchgD00002071	Efm0167	Blood culture	2011	No	Negative	4737374	2239023	74.77	ST017	15
wtchgD00002098	Efm0229	Blood culture	2013	No	Negative	5900840	2271662	75.86	ST017	16
wtchgD00002042	Efm0177	Blood culture	2012	No	Negative	3277686	2279635	76.12	ST017	17
wtchgD00002155	Efm0139	Blood culture	2011	No	Negative	4727788	2273256	75.91	ST017	18
wtchgD00002038	Efm0153	Blood culture	2011	Yes	Positive	2596522	2384209	79.62	ST017	19
wtchgD00002099	Efm0145	Blood culture	2011	No	Negative	3681096	2339746	78.13	ST017	20
wtchgD00002156	Efm0151	Blood culture	2011	Yes	Positive	4351744	2311091	77.17	ST017	21
wtchgD00002054	Efm0154	Blood culture	2011	No	Negative	5268308	2275095	75.97	ST017	22
wtchgD00002117	Efm0158	Blood culture	2011	No	Negative	4529570	2272473	75.88	ST017	23
wtchgD00002115	Efm0146	Blood culture	2011	No	Negative	4481284	2273565	75.92	ST017	24
wtchgD00002029	Efm0189	Blood culture	2012	No	Negative	2712136	2268794	75.76	ST017	25
wtchgD00002057	Efm0198	Blood culture	2012	No	Negative	5699978	2278741	76.09	ST017	26
wtchgD00002062	Efm0203	Blood culture	2012	No	Negative	4446470	2280986	76.17	ST017	27
wtchgD00002089	Efm0180	Blood culture	2012	Yes	Positive	4237000	2333391	77.92	ST017	28
wtchgD00002112	Efm0218	Blood culture	2012	Yes	Positive	5239412	2331625	77.86	ST017	29
wtchgD00002096	Efm0217	Blood culture	2012	Yes	Positive	6233762	2323726	77.60	ST017	30
wtchgD00002133	Efm0160	Blood culture	2011	Yes	Positive	3955624	2318068	77.41	ST017	31
wtchgD00002153	Efm0114	Blood culture	2006	No	Negative	4829014	2311723	77.19	ST018	32
wtchgD00002145	Efm0113	Blood culture	2006	No	Negative	4780704	2312171	77.21	ST018	33
wtchgD00002044	Efm0093	Blood culture	2004	No	Negative	4262444	2265917	75.67	ST018	34
wtchgD00002061	Efm0106	Blood culture	2005	No	Negative	4319806	2264786	75.63	ST018	35
wtchgD00002080	Efm0216	Blood culture	2012	No	Negative	3801624	2309816	77.13	ST262	36
wtchgD00002050	Efm0130	Blood culture	2007	No	Negative	5173584	2429892	81.14	ST233	37
wtchgD00002152	Efm0102	Blood culture	2004	No	Negative	4329342	2379789	79.47	ST017	38
wtchgD00002081	Efm0132	Blood culture	2007	No	Negative	3540862	2327803	77.73	ST117	39
wtchgD00002034	Efm0128	Blood culture	2007	Yes	Positive	3652926	2374484	79.29	ST990	40
wtchgD00002146	Efm0125	Blood culture	2007	Yes	Positive	4085870	2375709	79.33	ST990	41
wtchgD00002030	Efm0104	Blood culture	2004	No	Negative	3194502	2340830	78.17	ST017	42
wtchgD00002075	Efm0095	Blood culture	2004	No	Negative	4968166	2381213	79.52	ST017	43
wtchgD00002055	Efm0223	Blood culture	2013	No	Negative	5449274	2428926	81.11	ST080	44
wtchgD00002053	Efm0211	Blood culture	2012	No	Negative	5760852	2387376	79.72	ST080	45
wtchgD00002076	Efm0192	Blood culture	2012	No	Negative	4142256	2393646	79.93	ST080	46

wtchgD00002035	Efm0225	Blood culture	2013	No	Negative	5897656	2371957	79.21	ST080	47
wtchgD00002060	Efm0191	Blood culture	2012	No	Negative	4543198	2378767	79.43	ST555	48
wtchgD00002031	Efm0201	Blood culture	2012	Yes	Positive	4368982	2452881	81.91	ST078	49
wtchgD00002043	Efm0199	Blood culture	2012	Yes	Positive	4148408	2452436	81.89	ST078	50
wtchgD00002073	Efm0179	Blood culture	2012	Yes	Positive	3977232	2471052	82.52	ST341	51
wtchgD00002143	Efm0185	Blood culture	2012	Yes	Positive	3709568	2421075	80.85	ST341	52
wtchgD00002165	Efm0176	Blood culture	2012	Yes	Positive	4042238	2420950	80.84	ST991	53
wtchgD00002058	Efm0210	Blood culture	2012	Yes	Positive	5207014	2430556	81.16	ST341	54
wtchgD00002049	Efm0214	Blood culture	2012	Yes	Positive	5095280	2430223	81.15	ST341	55
wtchgD00002103	Efm0169	Blood culture	2011	Yes	Positive	3505432	2420710	80.83	ST341	56
wtchgD00002086	Efm0196	Blood culture	2012	Yes	Positive	5302084	2431163	81.18	ST341	57
wtchgD00002090	Efm0220	Blood culture	2012	Yes	Positive	4280182	2430153	81.15	ST341	58
wtchgD00002140	Efm0149	Blood culture	2011	Yes	Positive	4270178	2422775	80.90	ST341	59
wtchgD00002052	Efm0142	Blood culture	2011	No	Negative	4960698	2399000	80.11	ST252	60
wtchgD00002045	Efm0190	Blood culture	2012	No	Negative	5094332	2399869	80.14	ST252	61
wtchgD00002126	Efm0171	Blood culture	2011	No	Negative	4844904	2399546	80.13	ST252	62
wtchgD00002150	Efm0174	Blood culture	2011	Yes	Positive	4159982	2343371	78.25	ST252	63
wtchgD00002158	Efm0175	Blood culture	2012	Yes	Positive	4763402	2432079	81.21	ST341	64
wtchgD00002125	Efm0159	Blood culture	2011	Yes	Positive	4711244	2458588	82.10	ST414	65
wtchgD00002163	Efm0152	Blood culture	2011	Yes	Positive	3990756	2458298	82.09	ST414	66
wtchgD00002056	Efm0166	Blood culture	2011	Yes	Positive	4350288	2423289	80.92	ST341	67
wtchgD00002047	Efm0202	Blood culture	2012	Yes	Positive	5650236	2432997	81.24	ST341	68
wtchgD00002118	Efm0170	Blood culture	2011	Yes	Positive	3554164	2406199	80.35	ST341	69
wtchgD00002132	Efm0148	Blood culture	2011	Yes	Positive	4956032	2423321	80.92	ST341	70
wtchgD00002148	Efm0150	Blood culture	2011	Yes	Positive	3254244	2429053	81.11	ST341	71
wtchgD00002162	Efm0140	Blood culture	2011	Yes	Positive	4512894	2390217	79.82	ST341	72
wtchgD00002104	Efm0207	Blood culture	2012	No	Negative	5180208	2512310	83.89	ST203	73
wtchgD00002164	Efm0164	Blood culture	2011	No	Negative	3474648	2512054	83.88	ST203	74
wtchgD00002102	Efm0195	Blood culture	2012	No	Negative	3716328	2512076	83.89	ST203	75
wtchgD00002040	Efm0165	Blood culture	2011	No	Negative	4365366	2511843	83.88	ST203	76
wtchgD00002078	Efm0204	Blood culture	2012	No	Negative	5009590	2512527	83.90	ST203	77
wtchgD00002087	Efm0168	Blood culture	2011	No	Negative	4114298	2512838	83.91	ST203	78
wtchgD00002135	Efm0184	Blood culture	2012	No	Negative	4469040	2512784	83.91	ST203	79
wtchgD00002141	Efm0161	Blood culture	2011	No	Negative	3925882	2512461	83.90	ST203	80
wtchgD00002151	Efm0186	Blood culture	2012	No	Negative	4734862	2512923	83.91	ST203	81
wtchgD00002166	Efm0188	Blood culture	2012	No	Negative	2200148	2512132	83.89	ST203	82
wtchgD00002110	Efm0206	Blood culture	2012	No	Negative	3279842	2511772	83.88	ST203	83
wtchgD00002142	Efm0173	Blood culture	2012	No	Negative	3243984	2518013	84.08	ST203	84
wtchgD00002127	Efm0183	Blood culture	2012	No	Negative	3431158	2518827	84.11	ST203	85
wtchgD00002066	Efm0227	Blood culture	2013	No	Negative	4788350	2512383	83.90	ST203	86
wtchgD00002037	Efm0200	Blood culture	2012	No	Negative	3527236	2527460	84.40	ST203	87
wtchgD00002106	Efm0219	Blood culture	2012	No	Negative	4299346	2487997	83.08	ST203	88
wtchgD00002082	Efm0228	Blood culture	2013	No	Negative	4741482	2486285	83.02	ST203	89
wtchgD00002157	Efm0163	Blood culture	2011	No	Negative	5108048	2539494	84.80	ST203	90
wtchgD00002124	Efm0147	Blood culture	2011	No	Negative	3954910	2545457	85.00	ST203	91
wtchgD00002085	Efm0156	Blood culture	2011	Yes	Positive	3982278	2492999	83.25	ST078	92
wtchgD00002036	Efm0141	Blood culture	2011	Yes	Positive	3327096	2541346	84.86	ST078	93

wtchgD00002105	Efm0181	Blood culture	2012	Yes	Positive	3421552	2548604	85.10	ST203	94
wtchgD00002039	Efm0212	Blood culture	2012	No	Negative	3842126	2557608	85.41	ST203	95
wtchgD00002069	Efm0155	Blood culture	2011	Yes	Positive	4056592	2582911	86.25	ST203	96
wtchgD00002131	Efm0136	Blood culture	2007	No	Negative	4586176	2516350	84.03	ST203	97
wtchgD00002051	Efm0226	Blood culture	2013	No	Negative	6684914	2384388	79.62	ST017	98
wtchgD00002088	Efm0208	Blood culture	2012	Yes	Positive	5114794	2593201	86.59	ST192	99
wtchgD00002111	Efm0121	Blood culture	2007	Yes	Positive	3892196	2583125	86.26	ST203	100
wtchgD00002138	Efm0124	Blood culture	2007	Yes	Positive	3524992	2583029	86.25	ST203	101
wtchgD00002130	Efm0123	Blood culture	2007	Yes	Positive	3566890	2631317	87.87	ST203	102
wtchgD00002079	Efm0119	Blood culture	2006	Yes	Positive	3165842	2528204	84.42	ST017	103
wtchgD00002123	Efm0135	Blood culture	2007	Yes	Positive	5144942	2485718	83.00	ST017	104
wtchgD00002091	Efm0096	Blood culture	2004	Yes	Positive	4149970	2562147	85.56	ST203	105
wtchgD00002028	Efm0092	Blood culture	2003	No	Negative	3387272	2536237	84.69	ST203	106
wtchgD00002093	Efm0108	Blood culture	2005	Yes	Positive	3225388	2568682	85.78	ST203	107
wtchgD00002109	Efm0109	Blood culture	2005	Yes	Positive	4287942	2569244	85.79	ST203	108
wtchgD00002113	Efm0134	Blood culture	2005	Yes	Positive	5245794	2572345	85.90	ST203	109
wtchgD00002161	Efm0127	Blood culture	2007	Yes	Positive	3921108	2592997	86.59	ST203	110
wtchgD00002083	Efm0144	Blood culture	2011	Yes	Positive	3217408	2604167	86.96	ST203	111
wtchgD00002063	Efm0118	Blood culture	2006	No	Negative	3506442	2535867	84.68	ST203	112
wtchgD00002122	Efm0122	Blood culture	2007	Yes	Positive	4240316	2565687	85.68	ST203	113
wtchgD00002137	Efm0112	Blood culture	2006	No	Negative	4200808	2535878	84.68	ST203	114
wtchgD00002128	Efm0099	Blood culture	2004	No	Negative	4472036	2536101	84.69	ST203	115
wtchgD00002077	Efm0107	Blood culture	2007	No	Negative	3670892	2535334	84.66	ST203	116
wtchgD00002136	Efm0100	Blood culture	2004	No	Negative	3738440	2535633	84.67	ST203	117
wtchgD00002120	Efm0098	Blood culture	2004	No	Negative	4832900	2536013	84.68	ST203	118
wtchgD00002129	Efm0111	Blood culture	2005	No	Negative	2892180	2535683	84.67	ST203	119
wtchgD00002048	Efm0117	Blood culture	2006	No	Negative	3745058	2535228	84.66	ST203	120
wtchgD00002121	Efm0110	Blood culture	2005	Yes	Positive	4456736	2554821	85.31	ST203	121
wtchgD00002149	Efm0162	Blood culture	2011	No	Negative	4369836	2566822	85.71	ST203	122
wtchgD00002147	Efm0138	Blood culture	2011	No	Negative	2025366	2605310	87.00	ST203	123
wtchgD00002154	Efm0126	Blood culture	2007	No	Negative	4360104	2574448	85.97	ST203	124
wtchgD00002067	Efm0143	Blood culture	2011	No	Negative	3638724	2575029	85.99	ST203	125
wtchgD00002097	Efm0133	Blood culture	2007	No	Negative	5036258	2574353	85.96	ST203	126
wtchgD00002032	Efm0116	Blood culture	2006	No	Negative	3405482	2535107	84.65	ST203	127
wtchgD00002160	Efm0115	Blood culture	2006	No	Negative	3779648	2526245	84.36	ST203	128
wtchgD00002095	Efm0120	Blood culture	2006	No	Negative	3130464	2535509	84.67	ST203	129
wtchgD00002139	Efm0137	Blood culture	2007	No	Negative	3997940	2246177	75.01	ST682	130
wtchgD00002107	Efm0097	Blood culture	2004	No	Negative	3083806	2233646	74.59	ST032	131
wtchgD00002041	Efm0224	Blood culture	2013	No	Negative	4718050	2207245	73.71	ST092	132

Supplementary Table 2: Details of Novel MLSTs

Id	country	year	isolation_site	age_yr	van_gene	MLST	Housekeeping loci						
							atpA	ddl	gdh	purK	gyd	pstS	adk
wtchgD00002034	Australia	2007	blood	61	vanB	990	1	7	1	1	1	1	1
wtchgD00002037	Australia	2007	blood	61	vanB	990	1	7	1	1	1	1	1
wtchgD00002165	Australia	2012	blood	77	vanB	991	15	5	1	47	1	1	1

Supplementary Table 3: Details of Vancomycin resistant E. faecium bloodstream infection outbreaks

Isolate ID	MLST	Month	Year	Outbreak Number	Outbreak by MLST	Number of SNPs between isolates on WGS	Outbreak by WGS
**Outbreak 1**					yes		no
wtchgD00002109	ST203	August	2005	1.1		24 snps	
wtchgD00002121	ST203	August	2005	1.2			
**Outbreak 2**					yes		yes
wtchgD00002130	ST203	March	2007	2.1		6 snps	
wtchgD00002111	ST203	March	2007	2.2			
**Outbreak 3**					yes		no
wtchgD00002132	ST341	July	2011	3.1		43 snps	
wtchgD00002140	ST341	July	2011	3.2			
**Outbreak 4**					yes		no
wtchgD00002156	ST17	August	2011	4.1		13 snps	
wtchgD00002038	ST17	August	2011	4.2			
**Outbreak 5**					yes		no
wtchgD00002103	ST341	December	2011	5.1		41 snps	
wtchgD00002118	ST341	December	2011	5.2			
**Outbreak 6**					yes		yes
wtchgD00002043	ST78	July	2012	6.1		1 snp	
wtchgD00002031	ST78	July	2012	6.2			
**Outbreak 7**					yes		no
wtchgD00002158	ST341	August	2012	7.1		15 snps	
wtchgD00002058	ST341	August	2012	7.2			

MLST: Multi-Locus Sequence Typing; SNPs: Single nucleotide polymorphisms; WGS: Whole genome sequencing
A possible outbreak was deemed to have occurred when more than one VRE bacteraemia of the same MLST type occurred in a calendar month. Of the seven events identified only two were true outbreaks if WGS criteria were applied (a SNP difference of 10 or less) and corresponds with red highlighted outbreaks in Figure 1.

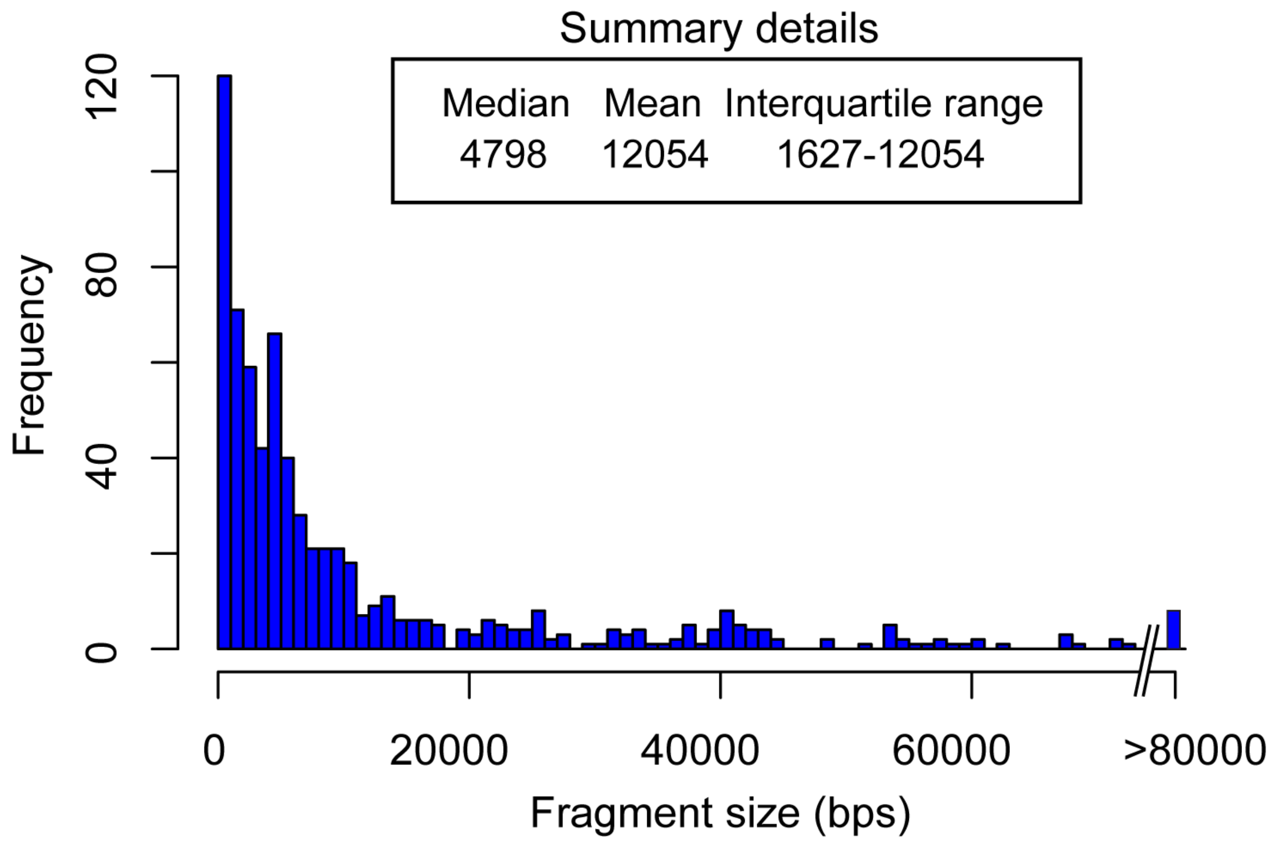

Supplementary Figure 1: Recombination fragments
Histogram of the estimated recombination fragments lengths predicted by ClonalFrameML. Summary details of recombination fragments in bps are shown in figure textbox. Of the 20 events ≥80kbps, three were greater than 100kbps with the largest fragment 279kbps.

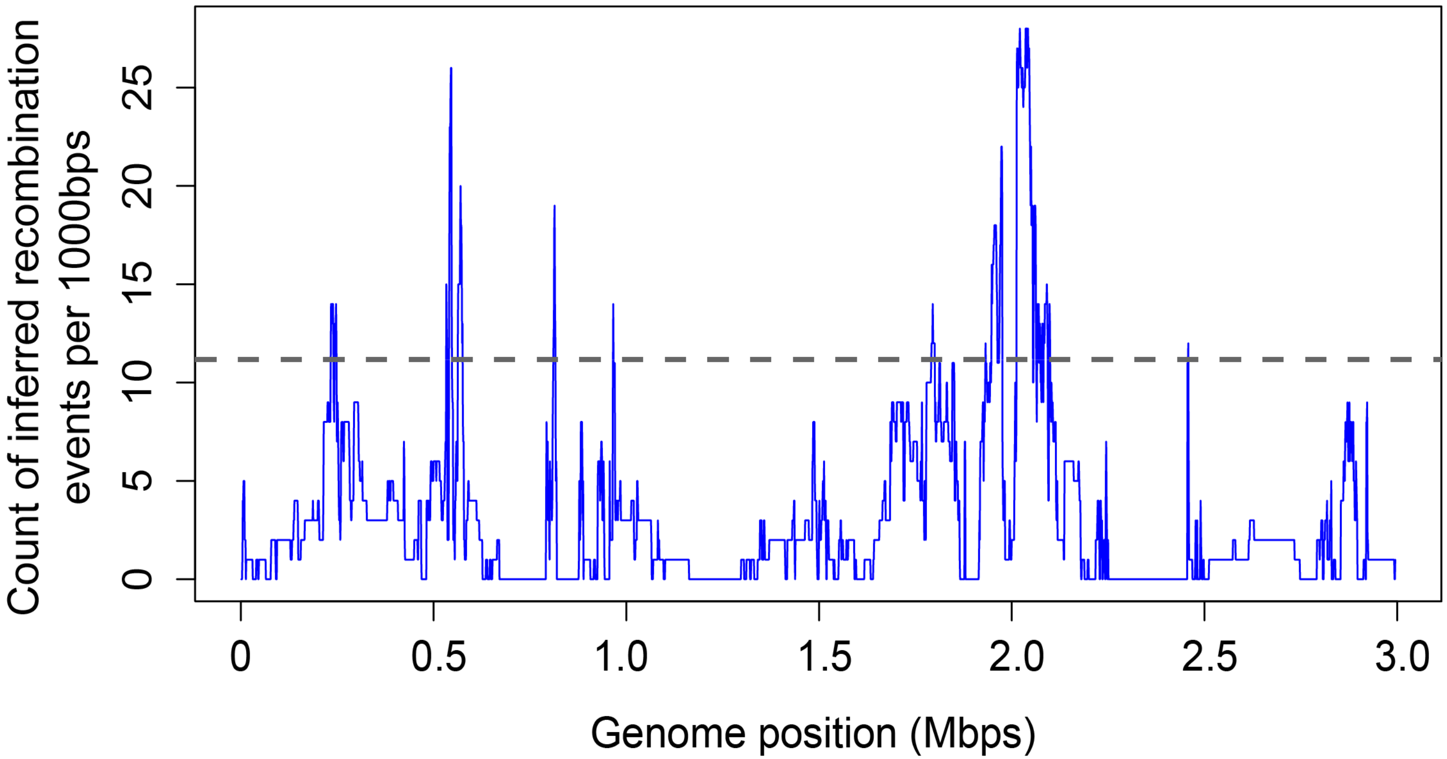

Supplementary Figure 2: Rates of recombination across genome
Manhattan plot of the number of recombination events across the genome per 1000bps. Dashed line represents the 95th percentile of events. Examination of potential 'hotspots' (i.e. areas of the genome with events above the 95th percentile) failed to show any enrichment for any specific pathway or genes including antimicrobial resistance genes.

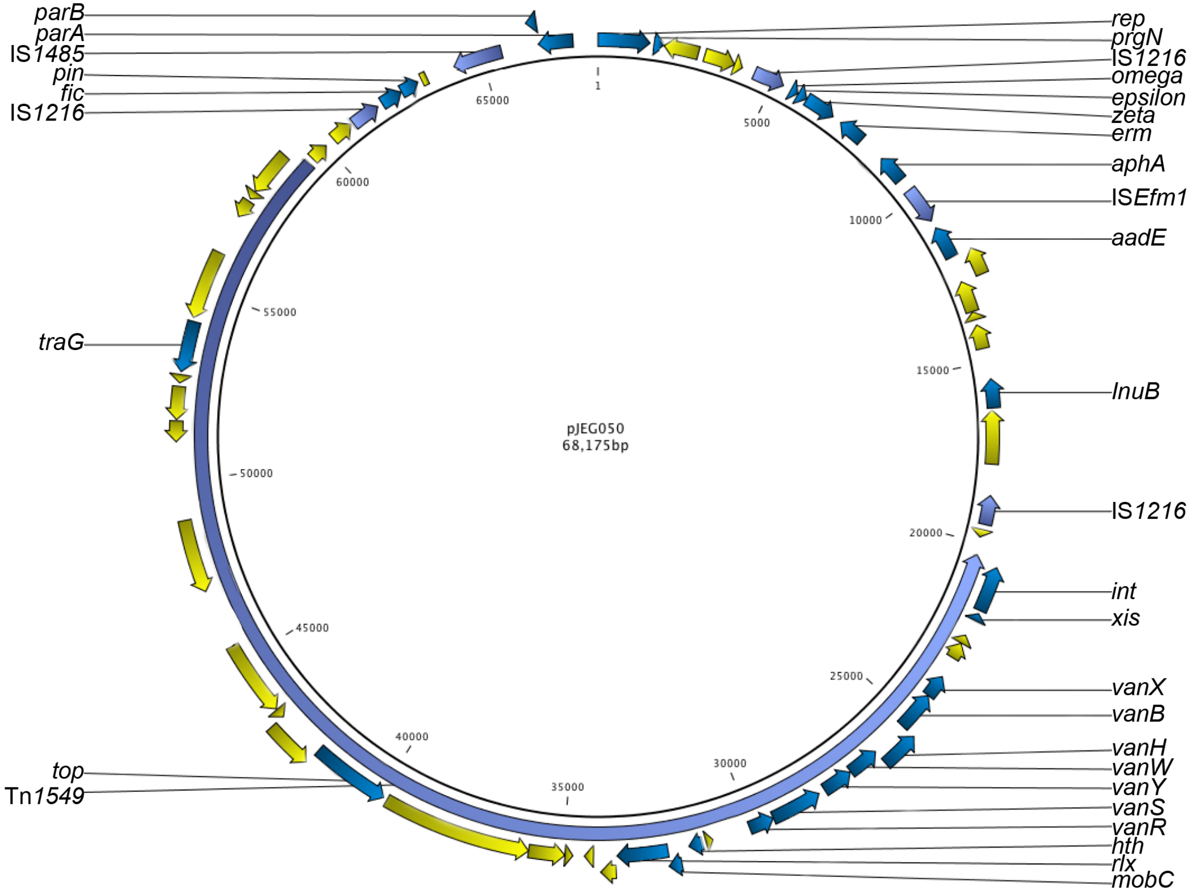

Supplementary Figure 3: Annotated plasmid pJEG050

Tn*1549* was inserted in the *yfbU* gene of an Aus0085 p3-like plasmid designated pJEG050 (GenBank acc. no.KR066794) and was detected in a single vancomycin-resistant *Enterococcus faecium* blood stream isolate.
